# Germline Mutation Enrichment in Pathways Controlling Endothelial Cell Homeostasis in Patients with Brain Arteriovenous Malformation: Implication for Molecular Diagnosis

**DOI:** 10.3390/ijms21124321

**Published:** 2020-06-17

**Authors:** Concetta Scimone, Francesca Granata, Marcello Longo, Enricomaria Mormina, Cristina Turiaco, Antonio A. Caragliano, Luigi Donato, Antonina Sidoti, Rosalia D’Angelo

**Affiliations:** 1 Department of Biomedical, Dental, Morphological and Functional Imaging Sciences, University of Messina, Via Consolare Valeria 1, 98125 Messina, Italy; cscimone@unime.it (C.S.); rdangelo@unime.it (R.D.); 2 Department of Biomolecular Strategies, Genetics and Avant-Garde Therapies, I.E.ME.S.T., Via Michele Miraglia, 90139 Palermo, Italy; 3 Neuroradiology Unit—Department of Biomedical, Dental, Morphological and Functional Imaging Sciences, University of Messina, Via Consolare Valeria 1, 98125 Messina, Italy; fgranata@unime.it (F.G.); mlongo@unime.it (M.L.); enricomaria.mormina@gmail.com (E.M.); cettinascimone.cs@gmail.com (C.T.); caraglia1987@gmail.com (A.A.C.); 4 Department of Clinical and Experimental Medicine, University of Messina, Consolare Valeria 1, 98125 Messina, Italy

**Keywords:** brain arteriovenous malformations, exome sequencing, endothelial properties, molecular diagnosis

## Abstract

Brain arteriovenous malformation (bAVM) is a congenital defect affecting brain microvasculature, characterized by a direct shunt from arterioles to venules. Germline mutations in several genes related to transforming growth factor beta (TGF-β)/BMP signaling are linked to both sporadic and hereditary phenotypes. However, the low incidence of inherited cases makes the genetic bases of the disease unclear. To increase this knowledge, we performed a whole exome sequencing on five patients, on DNA purified by peripheral blood. Variants were filtered based on frequency and functional class. Those selected were validated by Sanger sequencing. Genes carrying selected variants were prioritized to relate these genes with those already known to be linked to bAVM development. Most of the prioritized genes showed a correlation with the TGF-βNotch signaling and vessel morphogenesis. However, two novel pathways related to cilia morphogenesis and ion homeostasis were enriched in mutated genes. These results suggest novel insights on sporadic bAVM onset and confirm its genetic heterogeneity. The high frequency of germline variants in genes related to TGF-β signaling allows us to hypothesize bAVM as a complex trait resulting from the co-existence of low-penetrance loci. Deeper knowledge on bAVM genetics can improve personalized diagnosis and can be helpful with genotype–phenotype correlations.

## 1. Introduction

Brain arteriovenous malformations (bAVM, OMIM #108010) are vascular malformations affecting brain vasculature. Lack of a capillary bed and a direct shunt from arterioles to venules, as well as pericyte reduction, are characteristics of the lesions [1]. A vessel tangle is formed, usually called nidus. During transition from the feeding arteries to the nidus and then, to the draining veins, vessels show dysregulated differentiation patterns and severe enlargement. From arteries, blood perfuses to the nidus with high pressure, increasing risk of lesion rupture. Moreover, the mix of arterial and venous circulations within these lesions leads to a deficit in cerebral tissue oxygenation. This complex condition usually results in major clinical manifestations such as intracerebral hemorrhage and epileptic seizures, appearing in almost 50% of patients. Disease incidence is about 0.01% worldwide and usually arises at an early age [2]. Nevertheless, it most often occurs as a sporadic condition and only a few dozen cases are reported as inherited with an autosomal dominant pattern. Hereditary bAVM usually coexists with other vascular syndromes, such as Osler-Weber-Rendu syndrome, also known as hereditary hemorrhagic telangiectasia (HHT). HHT includes different phenotypes caused by mutations in genes related to the transforming growth factor beta 2 (TGF-βII) transduction pathway, such as *ENG*, *ACVRL1*, *SMAD4*, and *GDF2* [3,4,5]. Due to the severe remodeling rate and recidivism risk after total surgical resection, bAVMs are considered highly dynamic lesions. Therefore, lesion growth is now thought of as the result of continuous endogenous stimuli due to genetic factors. The low frequency of inherited bAVM makes molecular characterization of the disease difficult. Impaired expression of the ephrin family genes and of other vascular differentiation markers was reported by several authors [6,7]. At the same time, the sporadic nature of the disease can also be considered a result of numerous single nucleotide polymorphisms (SNPs) in genes involved in vasculogenesis and early angiogenesis pathways, as, for instance, in vascular endothelial growth factor (VEGF) and Notch signaling [8]. To improve knowledge on bAVM pathogenesis, we purified DNA from peripheral blood and performed a whole exome sequencing (WES) analysis on a group of five patients affected by sporadic bAVM. Then, we clustered genes carrying the detected mutations, highlighting pathways and prioritizing genes mainly linked to bAVM development.

## 2. Results

### 2.1. WES, Bioinformatic Analysis, and Filtering

An average of 81,170,229 million reads were output by the runs. Of these, 92.29% showed a Phred quality score > 30. After duplicate discard, an average of 75,411,435 reads were filtered and mapped to the GRCh38 human reference genome. The percentage of on-target reads was on average 71.57%, calculated on the deduplicated mapped read numbers. The quality report summary of each run is provided in Table S1. Regarding variant calling, a mean of 40,090 variants was annotated for each exome. Following the application of the above-mentioned filtering criteria, about 230 variants were selected for each sample. These included non-synonymous, nonsense, and frameshift mutations, whose reported minor allele frequency (MAF) was estimated to be < 0.01. Full lists are available in Table S2.

### 2.2. Gene Clustering and Prioritization

The ClueGO (Gene Ontology) enrichment analysis allowed to cluster the mutated genes for each exome in order to highlight the pathways they are involved in. In Table 1 only annotated terms showing the Bonferroni-adjusted *p*-value ≤ 0.05 were reported, both for each single term and for the entire cluster. The full lists are available in Table S3. Annotations related to cell adhesion (GO:1903392, GO:0051895, R-HSA:382054, GO:0038089, GO:0007157, GO:0120193, GO:1904886), cardiovascular development (WP:3668, GO:0003279, GO:0035904, GO:0060840, GO:0060976, GO:0060411), Notch signaling (GO:0007221, R-HSA:9021450, R-HSA:9013508, GO:0045747, WP:268, R-HSA:9012852), and TGFβR signaling (GO:1903846, GO:0030511) were highly represented in all the AVM samples. Furthermore, ontologies related to microtubule assembly and cell motility (GO:0003341, GO:0001578, GO:0001539, GO:0031122, GO:0060285) were also widely frequent. Interestingly, pathways related to ion conductance (GO:1904063, GO:0034766, GO:2001258, GO:0043267, GO:0016248, GO:0008200) were highly enriched in the AVM5 sample.

The table reports annotations from ClueGO enrichment analysis. For each sample, the enriched pathways are mentioned as GO Term (3rd column) and the ontology sources (GeneOntology, WikiPathways, Reactome) are reported (4th column), as well as the clustered genes (7th column). Only annotations showing Bonferroni-adjusted *p*-value ≤ 0.05 for term or group (5th and 6th columns, respectively) are reported. Full results are available in Table S3. ARFGAP: Adenosine diphosphate Ribosylation Factor-GTPase; AVM: Arteriovenous malformation. The number (1–5) indicates the sample; COPI: coating protein 1; GPCR: G protein-coupled receptor; MAP: Mitogen-Activated Protein; MAPK: Mitogen-Activated Protein Kinase; MKK: MAP Kinase Kinase; NCAM: Neural Cell Adhesion Molecule; PDGF: Platelet Derived Growth Factor; PLXND1: Plexin D1; TAK1: TGF-Beta-Activated Kinase 1; TNF: Tumor Necrosis Factor; IRE1: Inositol-Requiring Protein 1; v-SNARE: Vesicle-Soluble NSF (N-Ethylmaleimide-Sensitive Factor) Attachment Protein Receptor.Gene prioritization aimed to relate selected loci with others, already linked to bAVM development. ToppGene output a full list of the Test Gene Set, ordered according to their overall *p*-value. For each gene, the overall *p*-value is calculated on the basis of the single *p*-value for each training parameter considered. Therefore, also in this case, only genes showing an overall *p*-value ≤ 0.05 were selected for each sample. Together with this criterion, “Gene Ontology (GO) biological process” annotations were considered as prioritized for the five AVM exomes. These are *LTBP4*, *LTBP1*, *LRP1*, and *FBN2* for AVM1; *TAB1*, *RELN*, and *MAP2K3* for AVM2; *KDR* and *EPHA2* for AVM3; *NOTCH3*, *PLXND1*, *TAB1*, *CTBP2*, *SLIT2*, *RNF111*, *MAML1*, and *CHRNB2* for AVM4; and *AXIN1*, *EPHB2*, *DVL1*, and *CAMK2B* for AVM5 (Table 2). The complete lists with detailed statistical parameters as well as the annotations linking the prioritized genes to the training genes are available in Table S4.

Although prioritization analysis was quite exhaustive, further loci were considered. These added loci were selected as they are involved in vasculogenesis and carry missense and nonsense variants. In particular, they were *FLT4*, *NCoR2*, *CCN1*, and *GIMAP1* in the AVM2 sample; *NOTCH4* in AVM3; and *ENG* and *TGFBR2* in AVM5. In particular, *ENG* and *TGFRB2* are known to be highly linked to brain AVM and HHT development. *NCoR2* was affected by the novel nonsense variant c.2078G>T (p.Glu693Ter) (Ensembl Transcript ID: ENST00000405201.5). As a consequence, the mutated protein counts 692 amino acids rather than the 2514 amind acids of the wild-type one. The mutation was detected in a heterozygous condition and resulted as “Damaging” and “Disease causing” in SIFT (https://sift.bii.a-star.edu.sg/) [9] and MutationTaster (http://www.mutationtaster.org/) [10] prediction tools, respectively (not shown).

### 2.3. Sanger Validation

**Figure 1:**
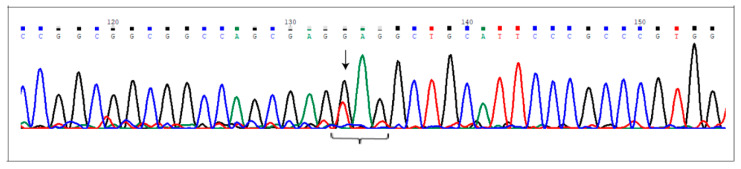
The c.2078G>T (p.Glu693Ter) at the *NCoR2* locus. Electropherogram shows the novel nonsense mutation affecting the *NCor2* gene, detected in heterozygous condition in AVM2 sample. Nucleotide substitution is indicated by the arrow. The brace indicates the DNA sequence corresponding to the triplet carrying the mutated nucleotide (the first one).

Table 3 lists the variants detected in the genes previously prioritized. All these variants were confirmed by Sanger sequencing and were not detected in our internal 10 control exomes obtained from healthy subjects. Figure 1 reports only the electropherogram of the novel nonsense variant, c.2078G>T (p.Glu693Ter) affecting the *NCoR2* locus.

## 3. Discussion

The genetic landscape of bAVM is to date quite elusive probably due to the very low frequency of inherited cases. Most patients are affected by sporadic forms whose molecular causes are waiting to be clarified. In many cases, lesions are congenital, increasing in size over the years until they become symptomatic. Due to the dynamic nature of bAVM and its high recidivism rate, it is well accepted that lesions arise as a consequence of continuous endogenous stimuli, as inherited and de novo genetic variants or epigenetic modifications occurring during embryo development [11,12]. Therefore, we performed WES analysis on a group of five patients affected by sporadic bAVM, highlighting the main pathways enriched by germline mutated genes. Despite mutated genes differing among patients, the pathways in which they converge are the same. Firstly, we checked for variants in *ENG*, *NOTCH4*, and *TGFβR2* that are known to be involved in arteriovenous malformation development [13,14]. Then, we searched for *KRAS* c.35G>T (p.Gly12Val) that was recurrently detected in bAVM patients [15] and none of them carried the mutation, as well as other variants within the gene. However, this was expected as our analysis was performed on DNA purified by peripheral blood. Mosaic-activating *KRAS* mutations, indeed, were found in sporadic AVM-derived specimens [16,17].

The ClueGO enrichment analysis revealed pathways related to microtubule formation, cell adhesion, and vascular remodeling as being highly enriched (Table 1). Moreover, prioritization analysis was performed for each sample to detect the main genes involved in TGFβR transduction pathways and, therefore, more likely associated to bAVM onset. As reported in Table S4a,b, several loci are noteworthy of consideration and the most relevant are listed in Table 2. However, among prioritized genes, here we briefly discuss those more likely related to bAVM development, in relation to the single sample. This selection was made considering significant *p*-values related to phenotypes and pathway, outputted by the ToppGene tool (Table S4a).

### 3.1. AVM1

Regarding AVM1, we focused on *LTBP1*, *LTBP4*, *FBN2*, and *LRP1* loci. *FBN2*, encoding for Fibrillin 2, and *LTBP1* and *LTBP4* belonging to the “latent transforming growth factor beta binding proteins” family, are ligands of TGF-β receptors [18,19]. With regard to *LRP1*, encoding for the LDL receptor related protein 1, expression data showed it is expressed in brain endothelial cells where it contributes to chemotactic cell migration, inducing sphingosine-1-phosphate proangiogenic signaling [20]. Moreover, depletion of *LRP1* determines defects of both large and small vessel morphogenesis leading to a lethal phenotype [21].

### 3.2. AVM2

Regarding data obtained from AVM2 exome, we considered variants affecting *TAB1*, *FLT4*, *RELN*, *MAP2K3*, *CCN1*, *GIMAP1*, and *NCoR2* loci. *TAB1* encodes for the TGF-β activated kinase 1 binding protein 1 and increases endothelial permeability, mediated by the non-canonical TGF-β pathway following inflammation stimuli [22]. Moreover, TAB1 activates the TAK1 kinase, an upstream modulator of the p38 MAPK signaling. *MAP2K3* is also involved [23] in the same pathway and, in particular, a physical interaction between TAK1 and MAP2K3 has been reported [24]. Involvement of inflammatory response in bAVM is, to date, well accepted [25,26] and in this context, we detected a nonsense mutation, the c.699G>A (p.Trp233Ter) in *GIMAP1* gene. Together with the TGFβR2 signaling, Notch transduction pathways were also reported as promoting AVM development [27]. Therefore, we also considered prioritizing the *NCor2* locus, encoding for nuclear receptor co-repressor. We found the novel nonsense mutation, c.2078G>T (p.Glu693Ter) (Figure 1). *FLT4*, instead, encodes for the vascular endothelial growth factor receptor 3 (VEGFR3) [28]. Finally, we considered *CCN1* locus, encoding for the cellular communication network factor 1. Its expression is increased in the extracellular matrix surrounding microvessels and is growth factor-inducible. The protein promotes integrin-mediated endothelial cell adhesion in response to mechanotransduction signaling [29]. This evidence is well congruent with bAVM pathogenesis due to frequent insult by the high blood pressure within the lesions.

### 3.3. AVM3

Together with *NOTCH4*, variants carried by *EPHA2*, *EPHB4*, and *KDR* were detected in sample AVM3. *EPHA2* and *EPHB2* encode for two proteins belonging to the ephrin family, a subgroup of protein-tyrosine kinase receptors. Ephrins are vessel differentiation markers and their role is pivotal during early vasculogenesis. In particular, mesenchymal cells express *EPHB2*, a feature of arterial morphogenesis. A model proposed by Adams et al. hypothesizes interaction among type-B ephrins differentially expressed in arteries and veins as the basis of a remodeling process that leads to sprouting and capillary network development [30]. *EPHB2* expression is upregulated in capillaries during inflammation. This results in increased endothelial permeability and loss of vessel differentiation [31]. The absence of EphA2, instead, was shown to impair the blood–brain barrier, resulting in inhibition of endothelial cell migration and in enhancement of tight junction formation in human brain microvascular endothelial cells (HBMECs) [32]. *KDR* encodes for the vascular VEGFR2, essential for the organization of the embryonic vasculature and angiogenic sprouting [33].

### 3.4. AVM4

The highest number of prioritized genes was in the AVM4 sample. GO annotations for biological process revealed involvement in vasculature morphogenesis for *NOTCH3*, *PLXND1*, *SLIT2*, and *MAML1* loci. At the embryo stage, VEGF and Notch transduction signaling modulates *PLXND1* expression to guide organ vasculogenesis by promoting endothelial cell migration and proliferation [34]. In adults, instead, *PLXND1* expression is physiologically low and limited to a few cell types, such as endothelial cells [35]. Balancing effects on migration were reported for *SLIT2* [36]. *MAML1* is described as a NOTCH coactivator, even if its role in angiogenesis needs more elucidation [37]. Regarding the TGF-β/BMP pathway, we focused on the *RNF111* gene, encoding for the E3 ubiquitin-protein ligase. One of its targets is the SMAD7 protein that acts by inhibiting TGF-β/BMP signaling. Therefore, RNF111 activity is required to promote SMAD7 degradation and to enhance the TGF-β/BMP pathway [38]. TGF-β signaling is also upregulated by increased levels of CTBP2 (C-terminal binding protein 2), driving endothelial-to-mesenchymal transition (EMT) [39]. This gene was also mutated in the AVM4 patient. In the end, we focused our attention on the GO terms regarding the response to hypoxia (GO:0001666, GO:0036293, GO:0070482) with the patient as a carrier of rs55685423 (c.1191G>C, p.Gln397His) in the *CHRNB2* locus. This gene encodes for the cholinergic receptor nicotinic beta 2 subunit [40]. Its expression was demonstrated in HBMECs, where it contributes to capillary network formation and to angiogenic response to inflammation [41]. Notably, the same ontologies were also found in the AVM3 patient, annotated by the *KDR* locus.

### 3.5. AVM5

Finally, in the AVM5 sample we identified two variants in *ENG* and *TGFBR2* loci, rs139398993 (c.392C>T, p.Pro131Leu) and rs35766612 (c.1159G>T, p.Val387Leu), respectively. Moreover, *EPHB2* was also affected by a missense variant. Based on human and mouse phenotype ontologies, *DVL1* and *AXIN1* were annotated to the “cerebrovascular disease” term and, in particular, with AVM and telangiectasia phenotypes. *DVL1* is known to control postnatal angiogenesis [42]. *AXIN1* encodes for a negative regulator of the Wnt pathway, also enhancing TGF-β signaling by promoting the degradation of the inhibitory SMAD7, in a RNF111-dependent manner [43]. Surprisingly, it was recently described as an important regulator of embryo central nervous system (CNS) angiogenesis, and overexpression leads to premature vascular regression, followed by progressive dilation and inhibition of vascular maturation [44].

### 3.6. Novel Insights 

Together with TGF-β/Notch signaling, GO annotations derived from the ClueGO enrichment analysis (Table 1) highlight a relevant presence of ontologies related to microtubule and cilia organization (GO:0003341, GO:0001578, GO:0031122, GO:0001539, GO:0060285). A recent study demonstrated that cilia are widely represented in endothelial cells during early vasculogenesis and in the later stages as vessel bifurcation point anastomosis. Zebrafish knock-down for cilia biogenesis gene models showed cilia disassembly following shear stress, resulting in remodeling of endothelial cell architecture and increased permeability and hemorrhagic events. Moreover, hemorrhages were only observed in head vasculature and were not observed in the trunk or caudal vessels [45]. Based on this evidence, germline defects in genes controlling cilia assembly might also contribute to brain AVM development as the result of mechanical stress induced by high blood flow and pressure. Clearly, this hypothesis needs to be adequately validated.

Another important property of the blood–brain barrier is the highly selective control of solute transport which is maintained by the exact spatial distribution of membrane transporters and ion channels. Polarization is a key factor for morpho-functional homeostasis of endothelial cells and was shown to be driven by VEGF via Ca^2+^ specific signaling pathways [46]. Moreover, dysregulation of K^+^ ion influx in non-excitable cells was shown to lead to hyperpolarization of membrane potential with consequent increased intracellular Ca^2+^. This results in enhancement of cell proliferation and was also demonstrated in brain capillary endothelial cells [47]. However, if physiological Ca^2+^ concentration is abnormally excessive, endothelial cells undergo apoptosis [48]. Therefore, we focused attention on GO terms from the ClueGO analysis of sample AVM5. As shown in Table 1, GO terms related to transmembrane ion transport (GO:1904063, GO:0034766, GO:2001258, GO:0043267, GO:0016248, GO:0008200) are largely enriched, as well as those related to membrane biogenesis (GO:0044091, GO:0071709, GO:1904886) making the hypothesis of misregulation in bAVM onset conceivable.

Although these are preliminary findings, they are in accordance with what was recently published by Hauer and colleagues. They describe dysregulated expression of genes also involving cytoskeleton network and transmembrane transport in bAVM-derived specimens, when compared to intracranial control arteries [49]. Therefore, these results allow to elicit other mechanisms in pathogenesis of bAVM not only confined to the canonical TGFβR2 pathway.

### 3.7. Final Considerations

**Figure 2:**
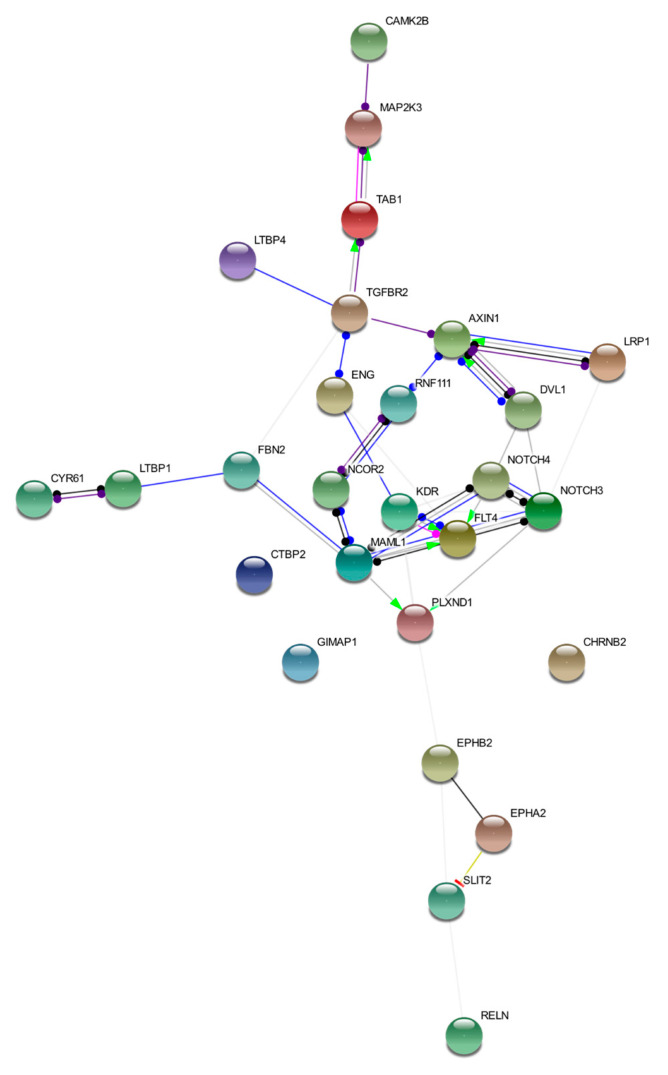
Functional network of prioritized genes. The image describes observed and inferred functional interactions linking prioritized genes. Nodes represent input proteins while edges represent protein–protein associations. Each edge color indicates a specific annotation. Green: activation; red: inhibition; blue: binding; light blue: phenotype; black: reaction; violet: catalysis; pink: posttranslational modification; yellow: transcriptional regulation.

We discussed loci affected by germline variants in five bAVM samples. Despite these loci differing among the samples, they converged in regulation of the same cellular signaling pathways. This interconnection is represented in Figure 2. The image was obtained by STRING tool Version 11.0 (https://string-db.org/) [50]. Details on nodes and edges are supplied in Table S5.

According to prioritized genes, our data support findings previously reported [51,52] and, in particular, the genetic heterogeneity of the disease. These results suggest that sporadic bAVM is probably not a monogenic condition, rather it arises during early vasculogenesis at the embryo stage. In particular, following fertilization, the combination of both inherited and eventually de novo genetic variants in numerous loci controlling vessel development could result in early vasculogenesis impairment and lesion onset. However, the evidence that these patients develop lesions only in the CNS underlines the importance of the cross-talk between glial cells and endothelium during neurodevelopment and blood–brain barrier morphogenesis. Most genes considered here show an early peculiar expression in neural progenitor cells that contributes to correct vasculogenesis and angiogenetic processes. In this context, proteins related to axon guidance such as Slits, plexins, and ephrins are exhaustive examples [53,54]. However, a last consideration regards genes involved in DNA repair such as *WRN*, *FANCC*, *BRCA2*, *TP53BP1*, and others carrying rare missense variants. These variants might cause protein functional alteration and, subsequently, DNA repair impairment. At the embryo stage, this might trigger DNA errors resulting in somatic mutations. Clearly, as our study focused on germline variants, the role of somatic mutations is not evaluated here.

Germline genetic variants are endogenous and permanent factors affecting both early vasculogenesis and late angiogenesis. Endothelial remodeling is a continuous phenomenon, and this can explain the increased recidivism rate of bAVM. Therefore, our hypothesis regards the possibility of considering bAVM as the result of the co-existence of numerous low-penetrance loci controlling different processes during endothelial cell differentiation and maturation. This idea is supported by two observations. We selected only loci affected by rare variants (MAF < 0.01) and then, those most likely related to rare disease onset. Rare variants at the same loci were searched for in our internal 10 control exomes obtained from healthy subjects and none was detected.

Clearly, the main limitation of the study is related to the few samples considered and, certainly, results require further validations on a larger patient cohort.

Despite this being a preliminary investigation, the possibility of detecting novel loci and germline mutations potentially involved in bAVM onset will allow to hypothesize a strategy for molecular diagnosis, preferably based on a panel of selected genes.

## 4. Materials and Methods

### 4.1. Patient Recruitment and WES Analysis

**Figure 3:**
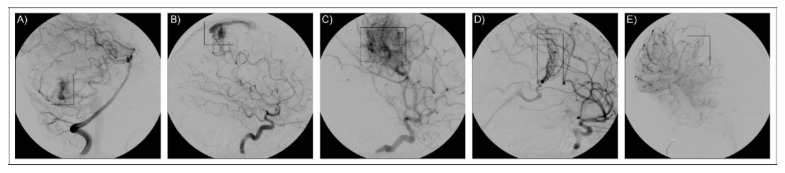
Imaging for neuroradiological diagnosis. The figure reports cerebral angiographies that confirm the presence of bAVM lesions. The five images refer to the five patients as reported in the text: (**A**) AVM1, (**B**) AVM2, (**C**) AVM3, (**D**) AVM4, (**E**) AVM5. AVM lesions are framed in the rectangular box.

The study was performed on a group of five Italian patients (AVM1–5) diagnosed with bAVM following cerebral angiography investigation (Figure 3). A severity lesion score was assigned to each patient based on the Spetzler–Martin grading system [55]. Anamnestic data are presented in Table 4. No familiar history of bAVM was reported for the patients and they were classified as sporadic. Patients were fully informed on their enrolment in the study and informed consent was obtained, and for underaged patients as well. DNA samples were collected from peripheral blood and purified by the QIAamp DNA Blood Mini Kit (Qiagen). Qualitative and quantitative measurements of the samples were performed by NanoDrop spectrophotometer (Thermo Fisher Scientific) and by a Qubit fluorometer (Thermo Fisher Scientific). Paired-end libraries were obtained by the SureSelect Human All Exon V7 (Agilent) kit and sequenced on a HiSeq 2500 Illumina platform.

### 4.2. Bioinformatic Analysis

FastQ data obtained from sequencing runs were quality checked by the FastQC (v.0.11.7) tool (http://www.bioinformatics.babraham.ac.uk/projects/fastqc). Only reads presenting a Phred score ≥ 28 were selected after trimming and aligned to the GRCh38 Human Reference Genome by the Burrows–Wheeler Aligner (BWA) algorithm [56]. Duplicate reads were removed by the MarkDuplicate tool provided by Picard toolkit (v.2.18.23) (“Picard Toolkit.” 2019. Broad Institute, GitHub Repository. http://broadinstitute.github.io/picard/; Broad Institute). Then, the Indel realignment and the base recalibration were performed by the Genome Analysis Toolkit (GATK) (v.4.1.3.0) (https://software.broadinstitute.org/gatk/). Variant calling was executed by FreeBayes [57], while ANNOVAR v.2018Apr16 [58] was used for variant annotation.

### 4.3. Variant Filtering Criteria

Before proceeding with downstream analysis, annotated genes and variants were filtered on the basis of several criteria. Variants showing quality score < 150 were discarded. This threshold value was established by the observation that several variants with depth lower than 150 were not confirmed by the following Sanger sequencing validation. Filtered variants were classified by functional class and intronic, synonymous, non-coding RNA, and untranslated regions affecting variants were discarded. Missense, nonsense, frameshift, and short indels presenting an MAF < 0.01 were selected. Rare variants were preferred based on the low worldwide incidence of bAVM. For the MAF-based filtering, the values reported in the Genome Aggregation Database (https://gnomad.broadinstitute.org/) [59] and in the 1000 Genomes phase 3 project [60] were considered. 

### 4.4. Gene Clustering and Prioritization 

To visualize and functionally group genes carrying filtered variants, the ClueGO plug-in of Cytoscape software was used for each sample [61]. Clustering was performed on the basis of the GO Biological Process, REACTOME and WikiPathways ontologies. Groups showing Bonferroni step down corrected *p*-value ≤ 0.05 were considered significant and therefore selected for the purpose. Genes within the chosen groups were added to the Test Gene Set in ToppGene (https://toppgene.cchmc.org/) [62], a web-based tool for prioritization of candidate genes based on functional similarity to a training gene list. The Training Gene Set group was made up of *ENG*, *ACVRL1*, *TGFBR2*, *SMAD4*, and *GDF2* genes, already known to be causative of HHT and of a few familiar bAVM cases without HHT. The training parameters selected were “GO:Biological Process”, “Human Phenotype”, “Mouse Phenotype”, “Pathway”, “PubMed”, “Interaction”, and “Disease”. Five different analyses were run, one for each exome data. Statistical parameters were calculated applying the Bonferroni correction, and *p*-values ≤ 0.05 were considered significant.

### 4.5. Sanger Validation

Variants carried by prioritized genes were validated by Sanger sequencing, next to polymerase chain reaction (PCR) amplification. Primer sequences and PCR conditions are available upon request. Sanger sequencing was carried out using the BigDyeTerminator^©^ v3.1 Cycle Sequencing Kit chemistry and run on a 3130xl Genetic Analyzer (Applied Biosystems, Thermo Fisher Scientific). Moreover, all variants here considered were further searched in an in-house exome-control dataset obtained by WES data, collected on a cohort of 10 Caucasian healthy subjects, heterogeneous for sex and age. The healthy condition was confirmed by computed tomography.

Patients agreed to be enrolled in the study and their informed consents were obtained. The manuscript does not contain information attributable to their identity. The study involves human participants and was approved by the local Ethics Committee “A.O.U. G. Martino”, N.11/2011 date of approval: 14 December.2011.

## 5. Conclusions

As knowledge on bAVM is still very elusive, we recruited a group of patients affected by sporadic bAVM and performed WES analysis. Clustering of genes which were affected by rare variants highlighted cytoskeleton impairment as well as defective ion conduction in endothelial cells. Therefore, we hypothesize perturbations at these pathways as possible mechanisms involved in bAVM pathogenesis. We prioritized genes more likely linked to lesion development as *FBN2*, *TAB1*, *NCoR2*, *SLIT2*, *RNF111*, *CAMK2B*, *EPHA2*, and *EPHB2*. Although to date no correlation has been reported between gene mutations and clinical phenotype, further characterization of pathways involved in bAVM development could provide a valid criterion to relate molecular features to clinical presentation. In particular, lesion site, bleeding risk, and patient outcome could represent valid prognostic factors linked to patient genotype.

## Figures and Tables

**Table 1:** ClueGO (Gene Ontology) enrichment analysis results.

Sample	GO ID	GO Term	Ontology Source	Term *p*-Value Corrected with Bonferroni Step Down	Group *p*-Value Corrected with Bonferroni Step Down	Associated Genes Found
AVM1	GO:0003341	cilium movement	GO_BiologicalProcess-EBI-UniProt-GOA_27.02.2019_00h00	0.00	0.00	(*ASPM, CFAP206, DNAH1, DNAH5, HYDIN, TEKT5*)
	GO:0035082	axoneme assembly	GO_BiologicalProcess-EBI-UniProt-GOA_27.02.2019_00h00	0.01	0.00	(*CFAP206, DNAH1, DNAH5, HYDIN, RP1L1*)
	R-HSA:2129379	molecules associated with elastic fibers	REACTOME_Pathways_27.02.2019	0.01	0.05	(*FBN2, FBN3, LTBP1, LTBP4*)
	GO:2000105	positive regulation of DNA-dependent DNA replication	GO_BiologicalProcess-EBI-UniProt-GOA_27.02.2019_00h00	0.01	0.14	(*CDC7, FLG, SYTL2*)
	GO:0097722	sperm motility	GO_BiologicalProcess-EBI-UniProt-GOA_27.02.2019_00h00	0.02	0.00	(*ASPM, CACNA1I, DNAH1, DNAH5, SLC22A16, TEKT5*)
	R-HSA:1566948	elastic fiber formation	REACTOME_Pathways_27.02.2019	0.02	0.05	(*FBN2, FBN3, LTBP1, LTBP4*)
	GO:0030317	flagellated sperm motility	GO_BiologicalProcess-EBI-UniProt-GOA_27.02.2019_00h00	0.02	0.00	(*ASPM, CACNA1I, DNAH1, DNAH5, SLC22A16, TEKT5*)
	GO:0018410	C-terminal protein amino acid modification	GO_BiologicalProcess-EBI-UniProt-GOA_27.02.2019_00h00	0.03	0.02	(*ASPM, LCMT2, SH2B1*)
	GO:0005044	scavenger receptor activity	GO_BiologicalProcess-EBI-UniProt-GOA_27.02.2019_00h00	0.03	0.03	(*LRP1, MEGF10, STAB1*)
	GO:1903078	positive regulation of protein localization to plasma membrane	GO_BiologicalProcess-EBI-UniProt-GOA_27.02.2019_00h00	0.03	0.03	(*CARD14, LRP1, NKD2*)
	WP:3668	hypothesized pathways in pathogenesis of cardiovascular disease	WikiPathways_27.02.2019	0.04	0.05	(*FBN2, FBN3, LTBP1*)
	GO:0001578	microtubule bundle formation	GO_BiologicalProcess-EBI-UniProt-GOA_27.02.2019_00h00	0.05	0.00	(*CFAP206, DNAH1, DNAH5, HYDIN, RP1L1*)
	GO:1903392	negative regulation of adherens junction organization	GO_BiologicalProcess-EBI-UniProt-GOA_27.02.2019_00h00	0.05	0.07	(*DLEC1, LRP1, TEP1*)
	GO:0051895	negative regulation of focal adhesion assembly	GO_BiologicalProcess-EBI-UniProt-GOA_27.02.2019_00h00	0.05	0.07	(*DLEC1, LRP1, TEP1*)
AVM2	GO:0031122	cytoplasmic microtubule organization	GO_BiologicalProcess-EBI-UniProt-GOA_27.02.2019_00h00	0.03	0.02	(*GOLGA4, KIF19, MBD1, PCM1, TUBGCP6*)
	GO:0042267	natural killer cell mediated cytotoxicity	GO_BiologicalProcess-EBI-UniProt-GOA_27.02.2019_00h00	0.03	0.08	(*LGALS9, LILRB1, PIK3R6*)
	GO:0070228	regulation of lymphocyte apoptotic process	GO_BiologicalProcess-EBI-UniProt-GOA_27.02.2019_00h00	0.03	0.09	(*IRS2, LGALS9, SLC39A10*)
	R-HSA:450346	activated human TAK1 phosphorylates MKK3/MKK6	REACTOME_Reactions_27.02.2019	0.04	0.00	(*MAP2K3, NOD1, TAB1*)
	GO:0061098	positive regulation of protein tyrosine kinase activity	GO_BiologicalProcess-EBI-UniProt-GOA_27.02.2019_00h00	0.05	0.02	(*AGXT, DGKQ, DOK7, ERCC6, RELN*)
	R-HSA:450302	activated TAK1 mediates p38 MAPK activation	REACTOME_Pathways_27.02.2019	0.06	0.00	(*MAP2K3, NOD1, TAB1*)
	R-HSA:448424	interleukin-17 signaling	REACTOME_Pathways_27.02.2019	0.06	0.00	(*MAP2K3, NOD1, TAB1*)
	R-HSA:382054	PDGF binds to extracellular matrix proteins	REACTOME_Reactions_27.02.2019	0.07	0.04	(*COL6A3, COL6A6, SPP1*)
	WP:231	TNF alpha signaling pathway	WikiPathways_27.02.2019	0.08	0.00	(KSR1, MAP2K3, NFKBIE, NSMAF, TAB1)
	R-HSA:186797	signaling by PDGF	REACTOME_Pathways_27.02.2019	0.08	0.04	(*COL6A3, COL6A6, GRB7, SPP1*)
	GO:0036498	IRE1-mediated unfolded protein response	GO_BiologicalProcess-EBI-UniProt-GOA_27.02.2019_00h00	0.08	0.04	(*ARFGAP3, EXTL1, NR1H4, VAPB, WFS1*)
	GO:0035176	social behavior	GO_BiologicalProcess-EBI-UniProt-GOA_27.02.2019_00h00	0.09	0.02	(*HTT, MBD1, PCM1*)
	R-HSA:373739	ankyrins link voltage-gated sodium and potassium channels to spectrin and L1	REACTOME_Reactions_27.02.2019	0.10	0.05	(*ANK2, SCN7A, SPTA1*)
	R-HSA:445095	interaction between L1 and ankyrins	REACTOME_Pathways_27.02.2019	0.11	0.05	(*ANK2, SCN7A, SPTA1*)
	GO:1901618	organic hydroxy compound transmembrane transporter activity	GO_BiologicalProcess-EBI-UniProt-GOA_27.02.2019_00h00	0.11	0.03	(*AQP7, HTT, SLC10A6*)
	GO:0006890	retrograde vesicle-mediated transport, Golgi to endoplasmic reticulum	GO_BiologicalProcess-EBI-UniProt-GOA_27.02.2019_00h00	0.13	0.05	(*ARFGAP3, CENPE, ERGIC1, HTT, TAPBP*)
	GO:0035036	sperm-egg recognition	GO_BiologicalProcess-EBI-UniProt-GOA_27.02.2019_00h00	0.13	0.05	(*CATSPER2, ZAN, ZP1*)
	GO:0021846	cell proliferation in forebrain	GO_BiologicalProcess-EBI-UniProt-GOA_27.02.2019_00h00	0.13	0.02	(*KIF14, MBD1, PCM1*)
	GO:0018195	peptidyl-arginine modification	GO_BiologicalProcess-EBI-UniProt-GOA_27.02.2019_00h00	0.13	0.05	(*NR1H4, PADI2, PRMT7*)
	GO:0002753	cytoplasmic pattern recognition receptor signaling pathway	GO_BiologicalProcess-EBI-UniProt-GOA_27.02.2019_00h00	0.15	0.00	(*ALPK1, DHX58, NOD1, TAB1*)
	R-HSA:6807875	ARFGAP, cargo, v-SNAREs, and p24 proteins bind nascent COPI complex	REACTOME_Reactions_27.02.2019	0.19	0.05	(*ANK2, ARFGAP3, SPTA1*)
	R-HSA:6807877	ARFGAPs stimulate ARF GTPase activity	REACTOME_Reactions_27.02.2019	0.19	0.05	(*ANK2, ARFGAP3, SPTA1*)
	R-HSA:450294	MAP kinase activation	REACTOME_Pathways_27.02.2019	0.20	0.00	(MAP2K3, NOD1, TAB1)
	R-HSA:375165	NCAM signaling for neurite out-growth	REACTOME_Pathways_27.02.2019	0.22	0.04	(*COL6A3, COL6A6, SPTA1*)
	R-HSA:2022090	assembly of collagen fibrils and other multimeric structures	REACTOME_Pathways_27.02.2019	0.22	0.04	(*COL6A3, COL6A6, LAMA3*)
	R-HSA:168643	nucleotide-binding domain, leucine rich repeat containing receptor (NLR) signaling pathways	REACTOME_Pathways_27.02.2019	0.23	0.00	(*NOD1, PSTPIP1, TAB1*)
	GO:1903573	negative regulation of response to endoplasmic reticulum stress	GO_BiologicalProcess-EBI-UniProt-GOA_27.02.2019_00h00	0.23	0.04	(*NR1H4, PRKN, WFS1*)
AVM3	GO:0038089	positive regulation of cell migration by vascular endothelial growth factor signaling pathway	GO_BiologicalProcess-EBI-UniProt-GOA_27.02.2019_00h00	0.01	0.17	(*KDR, MYO1C, PKD1*)
	GO:0007157	heterophilic cell–cell adhesion via plasma membrane cell adhesion molecules	GO_BiologicalProcess-EBI-UniProt-GOA_27.02.2019_00h00	0.04	0.09	(*HMCN1, RP9, SPG7*)
	GO:0120193	tight junction organization	GO_BiologicalProcess-EBI-UniProt-GOA_27.02.2019_00h00	0.04	0.09	(*EPHA2, MYO1C, PDZD4*)
	GO:0032688	negative regulation of interferon-beta production	GO_BiologicalProcess-EBI-UniProt-GOA_27.02.2019_00h00	0.04	0.08	(*LRRFIP1, MYO1C, NLRC3*)
	GO:0048739	cardiac muscle fiber development	GO_BiologicalProcess-EBI-UniProt-GOA_27.02.2019_00h00	0.08	0.05	(*MYOM2, OBSL1, SPG7*)
AVM4	GO:0001539	cilium or flagellum-dependent cell motility	GO_BiologicalProcess-EBI-UniProt-GOA_27.02.2019_00h00	0.00	0.00	(*DNAH11, DNAH14, DNAH2, DNAH3, DNAH8, GAS8*)
	GO:0060285	cilium-dependent cell motility	GO_BiologicalProcess-EBI-UniProt-GOA_27.02.2019_00h00	0.00	0.00	(*DNAH11, DNAH14, DNAH2, DNAH3, DNAH8, GAS8*)
	GO:0007221	positive regulation of transcription of Notch receptor target	GO_BiologicalProcess-EBI-UniProt-GOA_27.02.2019_00h00	0.00	0.00	(*MAML1, NOTCH3, OPN1LW, PLXND1, SPHKAP*)
	WP:334	GPCRs, class B secretin-like	WikiPathways_27.02.2019	0.01	0.10	(*ADCYAP1R1, ADGRG2, GLP2R, SCTR*)
	GO:0003279	cardiac septum development	GO_BiologicalProcess-EBI-UniProt-GOA_27.02.2019_00h00	0.01	0.00	(*CRELD1, DNAH11, LRP2, MAML1, PLXND1, SLIT2, SMO, TAB1*)
	GO:0071503	response to heparin	GO_BiologicalProcess-EBI-UniProt-GOA_27.02.2019_00h00	0.02	0.01	(*AOC1, GPIHBP1, SLIT2*)
	R-HSA:9021450	PLXND1 gene expression is stimulated by NOTCH1/NOTCH3 coactivator complexes	REACTOME_Reactions_27.02.2019	0.02	0.00	(*MAML1, NOTCH3, PLXND1*)
	GO:0061476	response to anticoagulant	GO_BiologicalProcess-EBI-UniProt-GOA_27.02.2019_00h00	0.03	0.01	(*AOC1, GPIHBP1, SLIT2*)
	R-HSA:2162123	synthesis of prostaglandin (PG) and thromboxane (TX)	REACTOME_Pathways_27.02.2019	0.03	0.19	(*AKR1C3, HPGD, TBXAS1*)
	GO:0003205	cardiac chamber development	GO_BiologicalProcess-EBI-UniProt-GOA_27.02.2019_00h00	0.04	0.00	(*CRELD1, DNAH11, LRP2, MAML1, PKD1, PLXND1, SLIT2, SMO, TAB1*)
	GO:0048278	vesicle docking	GO_BiologicalProcess-EBI-UniProt-GOA_27.02.2019_00h00	0.05	0.15	(*CTBP2, SCFD2, STX11*)
	GO:0007157	heterophilic cell–cell adhesion via plasma membrane cell adhesion molecules	GO_BiologicalProcess-EBI-UniProt-GOA_27.02.2019_00h00	0.05	0.15	(*FAT4, HMCN1, NUFIP2*)
	GO:0021795	cerebral cortex cell migration	GO_BiologicalProcess-EBI-UniProt-GOA_27.02.2019_00h00	0.05	0.15	(*SLIT2, SRGAP2, SUN1*)
	GO:0002753	cytoplasmic pattern recognition receptor signaling pathway	GO_BiologicalProcess-EBI-UniProt-GOA_27.02.2019_00h00	0.05	0.25	(*ALPK1, IRAK2, TAB1*)
	GO:0007616	long-term memory	GO_BiologicalProcess-EBI-UniProt-GOA_27.02.2019_00h00	0.09	0.04	(*CTNS, GRIA1, PJA2, PRNP*)
	GO:0007613	memory	GO_BiologicalProcess-EBI-UniProt-GOA_27.02.2019_00h00	0.11	0.04	(*ADGRF1, CHRNB2, CIC, CTNS, GRIA1, PJA2, PRNP*)
	R-HSA:9013508	NOTCH3 intracellular domain regulates transcription	REACTOME_Pathways_27.02.2019	0.14	0.00	(*MAML1, NOTCH3, PLXND1*)
	GO:0035904	aorta development	GO_BiologicalProcess-EBI-UniProt-GOA_27.02.2019_00h00	0.25	0.00	(*LRP2, PLXND1, TAB1*)
	GO:0060840	artery development	GO_BiologicalProcess-EBI-UniProt-GOA_27.02.2019_00h00	0.25	0.00	(*HPGD, LRP2, NOTCH3, PLXND1, TAB1*)
	GO:0045747	positive regulation of Notch signaling pathway	GO_BiologicalProcess-EBI-UniProt-GOA_27.02.2019_00h00	0.27	0.00	(*MAML1, NEPRO, OPN1LW, SPHKAP*)
	GO:0035082	axoneme assembly	GO_BiologicalProcess-EBI-UniProt-GOA_27.02.2019_00h00	0.33	0.00	(*DNAH8, GAS8, RSPH1*)
	WP:268	Notch signaling	WikiPathways_27.02.2019	0.39	0.00	(*CTBP2, MAML1, NOTCH3*)
	GO:1903846	positive regulation of cellular response to transforming growth factor beta stimulus	GO_BiologicalProcess-EBI-UniProt-GOA_27.02.2019_00h00	0.40	0.00	(*OPN1LW, RNF111, SPHKAP*)
	GO:0030511	positive regulation of transforming growth factor beta receptor signaling pathway	GO_BiologicalProcess-EBI-UniProt-GOA_27.02.2019_00h00	0.40	0.00	(*OPN1LW, RNF111, SPHKAP*)
	R-HSA:9012852	signalling by NOTCH3	REACTOME_Pathways_27.02.2019	0.41	0.00	(*MAML1, NOTCH3, PLXND1*)
	GO:0060976	coronary vasculature development	GO_BiologicalProcess-EBI-UniProt-GOA_27.02.2019_00h00	0.41	0.00	(*LRP2, PLXND1, TAB1*)
	GO:0060411	cardiac septum morphogenesis	GO_BiologicalProcess-EBI-UniProt-GOA_27.02.2019_00h00	0.42	0.00	(*DNAH11, LRP2, SLIT2, SMO*)
AVM5	WP:2572	primary focal segmental glomerulosclerosis (FSGS)	WikiPathways_27.02.2019	0.03	0.05	(*AGRN, CAMK2B, LAMB2, LMX1B, MKI67*)
	GO:0006027	glycosaminoglycan catabolic process	GO_BiologicalProcess-EBI-UniProt-GOA_27.02.2019_00h00	0.04	0.08	(*AGRN, CD44, GPX4*)
	GO:0031952	regulation of protein autophosphorylation	GO_BiologicalProcess-EBI-UniProt-GOA_27.02.2019_00h00	0.08	0.04	(*CA5A, CAMK2B, ENG*)
	GO:1904886	beta-catenin destruction complex disassembly	GO_BiologicalProcess-EBI-UniProt-GOA_27.02.2019_00h00	0.08	0.05	(*AXIN1, CA5A, DVL1*)
	GO:0097150	neuronal stem cell population maintenance	GO_BiologicalProcess-EBI-UniProt-GOA_27.02.2019_00h00	0.09	0.05	(*BRCA2, FANCC, MBD1*)
	R-HSA:2142753	arachidonic acid metabolism	REACTOME_Pathways_27.02.2019	0.09	0.05	(*CYP4B1, CYP4F3, GPX4, PRXL2B*)
	GO:1904063	negative regulation of cation transmembrane transport	GO_BiologicalProcess-EBI-UniProt-GOA_27.02.2019_00h00	0.10	0.04	(*ARHGEF40, CA5A, CAMK2B, EPHB2, KEL*)
	GO:0007528	neuromuscular junction development	GO_BiologicalProcess-EBI-UniProt-GOA_27.02.2019_00h00	0.15	0.05	(*AGRN, DVL1, LAMB2*)
	GO:0034766	negative regulation of ion transmembrane transport	GO_BiologicalProcess-EBI-UniProt-GOA_27.02.2019_00h00	0.18	0.04	(*ARHGEF40, CA5A, CAMK2B, EPHB2, KEL*)
	GO:0043267	negative regulation of potassium ion transport	GO_BiologicalProcess-EBI-UniProt-GOA_27.02.2019_00h00	0.22	0.04	(*CA5A, CPAMD8, KEL*)
	GO:0044091	membrane biogenesis	GO_BiologicalProcess-EBI-UniProt-GOA_27.02.2019_00h00	0.25	0.04	(*CA5A, EPHB2, PTPRH*)
	GO:2001258	negative regulation of cation channel activity	GO_BiologicalProcess-EBI-UniProt-GOA_27.02.2019_00h00	0.26	0.04	(*CA5A, CAMK2B, EPHB2*)
	GO:0071709	membrane assembly	GO_BiologicalProcess-EBI-UniProt-GOA_27.02.2019_00h00	0.28	0.04	(*CA5A, EPHB2, PTPRH*)
	GO:0016248	channel inhibitor activity	GO_BiologicalProcess-EBI-UniProt-GOA_27.02.2019_00h00	0.28	0.04	(*CA5A, CAMK2B, PHPT1*)
	GO:0008200	ion channel inhibitor activity	GO_BiologicalProcess-EBI-UniProt-GOA_27.02.2019_00h00	0.28	0.04	(*CA5A, CAMK2B, PHPT1*)

The table reports annotations from ClueGO enrichment analysis. For each sample, the enriched pathways are mentioned as GO Term (3rd column) and the ontology sources (GeneOntology, WikiPathways, Reactome) are reported (4th column), as well as the clustered genes (7th column). Only annotations showing Bonferroni-adjusted *p*-value ≤ 0.05 for term or group (5th and 6th columns, respectively) are reported. Full results are available in Table S3. ARFGAP: Adenosine diphosphate Ribosylation Factor-GTPase; AVM: Arteriovenous malformation. The number (1–5) indicates the sample; COPI: coating protein 1; GPCR: G protein-coupled receptor; MAP: Mitogen-Activated Protein; MAPK: Mitogen-Activated Protein Kinase; MKK: MAP Kinase Kinase; NCAM: Neural Cell Adhesion Molecule; PDGF: Platelet Derived Growth Factor; PLXND1: Plexin D1; TAK1: TGF-Beta-Activated Kinase 1; TNF: Tumor Necrosis Factor; IRE1: Inositol-Requiring Protein 1; v-SNARE: Vesicle-Soluble NSF (N-Ethylmaleimide-Sensitive Factor) Attachment Protein Receptor.

**Table 2:** Genes prioritized by ToppGene tool.

Ontology	Feature	ID	Name	Genes
*GO: Biological Process*	Vessel development	GO:0001525	Angiogenesis	*ACVRL1 ENG **EPHA2 EPHB2** GDF2 **KDR NOTCH3 PLXND1 SLIT2** TGFBR2*
		GO:0001568	Blood vessel development	*ACVRL1 ENG **EPHA2 EPHB2** GDF2 **KDR PKD1 LRP1 LTBP1 TAB1 HPGD LRP2 NOTCH3 PLXND1 SLIT2 SMO** TGFBR2*
		GO:0001569	Branching involved in blood vessel morphogenesis	*ENG GDF2 **PLXND1** TGFBR2*
		GO:0001570	Vasculogenesis	*ENG **EPHA2** GDF2 **SMO KDR** TGFBR2*
		GO:0001944	Vasculature development	*ACVRL1 ENG **EPHA2** GDF2 **KDR PKD1 LRP1 LTBP1 HPGD LRP2 NOTCH3 PLXND1 SLIT2 SMO TAB1** TGFBR2*
		GO:0048514	Blood vessel morphogenesis	*ACVRL1 ENG **EPHA2 EPHB2** GDF2 **LRP1 KDR HPGD LRP2 NOTCH3 PLXND1 SLIT2 SMO** TGFBR2*
		GO:1901342	Regulation of vasculature development	*ACVRL1 ENG **EPHA2** GDF2 **KDR PLXND1** TGFBR2*
	TGFBR signaling	GO:0007179	Transforming growth factor beta receptor signaling pathway	*ACVRL1 **AXIN1** ENG **FBN2** GDF2 **LTBP1 LTBP4 HPGD RNF111** SMAD4 **TAB1** TGFBR2*
		GO:0017015	Regulation of transforming growth factor beta receptor signaling pathway	***AXIN1** ENG **FBN2** GDF2 **LTBP1 LTBP4 RNF111** SMAD4 TGFBR2*
		GO:0071559	Response to transforming growth factor beta	*ACVRL1 **AXIN1** ENG **FBN2** GDF2 **HPGD RNF111 LTBP1 LTBP4** SMAD4 **TAB1** TGFBR2*
		GO:0071560	Cellular response to transforming growth factor beta stimulus	*ACVRL1 **AXIN1** ENG **FBN2** GDF2 **LTBP1 LTBP4 HPGD RNF111** SMAD4 **TAB1** TGFBR2*
		GO:1903844	Regulation of cellular response to transforming growth factor beta stimulus	***AXIN1** ENG **FBN2** GDF2 **LTBP1 LTBP4** SMAD4 TGFBR2*
	Heart development	GO:0003007	Heart morphogenesis	*ACVRL1 **DNAH11** ENG SMAD4 **FAT4 LRP2 PLXND1 SLIT2 SMAD4 SMO TAB1** TGFBR2*
		GO:0003205	Cardiac chamber development	***DNAH11** ENG **LRP2 MAML1 PLXND1 SLIT2 LRP1 LTBP1** SMAD4 **SMO TAB1** TGFBR2*
		GO:0003206	Cardiac chamber morphogenesis	***DNAH11** ENG **LRP2 SLIT2** SMAD4 **SMO** TGFBR2*
		GO:0003208	Cardiac ventricle morphogenesis	*ENG **LRP2** SMAD4 TGFBR2*
		GO:0003231	Cardiac ventricle development	*ENG **LTBP1 LRP2 SLIT2** SMAD4 TGFBR2*
		GO:0003279	Cardiac septum development	***DNAH11** ENG **LRP1 LTBP1 LRP2 MAML1 PLXND1 SLIT2** SMAD4 **SMO TAB1** TGFBR2*
		GO:0007507	Heart development	*ACVRL1 **DNAH5 DNAH11 DVL1** ENG **KDR PKD1 LRP1 LTBP1 FAT4 LRP2 MAML1 PLXND1 SLIT2** SMAD4 **SMO TAB1** TGFBR2*
		GO:0060411	Cardiac septum morphogenesis	***DNAH11** ENG **LRP2 SLIT2** SMAD4 **SMO** TGFBR2*
		GO:0072358	Cardiovascular system development	*ACVRL1 ENG **EPHA2 EPHB2** GDF2 **KDR PKD1 LRP1 LTBP1 HPGD LRP2 NOTCH3 PLXND1 SLIT2 SMO TAB1** TGFBR2*
		GO:0072359	Circulatory system development	*ACVRL1 **DNAH5 DNAH11 DVL1** ENG **EPHA2 EPHB2 FAT4** GDF2 **LRP1 LTBP1 KDR PKD1 HPGD LRP2 MAML1 NOTCH3 PLXND1 SLIT2** SMAD4 **SMO TAB1** TGFBR2*
		GO:2000826	Regulation of heart morphogenesis	*ENG SMAD4 **SMO** TGFBR2*
	BMP signaling	GO:0030509	BMP signaling pathway	*ACVRL1 ENG GDF2 **KDR LRP2** SMAD4*
		GO:0030510	Regulation of BMP signaling pathway	*ACVRL1 ENG GDF2 **KDR LRP2** SMAD4*
		GO:0030513	Positive regulation of BMP signaling pathway	*ACVRL1 ENG GDF2 **KDR** SMAD4*
		GO:0071772	Response to BMP	*ACVRL1 ENG GDF2 **KDR LRP2** SMAD4*
		GO:0071773	Cellular response to BMP stimulus	*ACVRL1 ENG GDF2 **KDR LRP2** SMAD4*
	Endothelial/mesenchymal differentiation	GO:0001935	Endothelial cell proliferation	*ACVRL1 ENG **EPHA2** GDF2 **KDR***
		GO:0001936	Regulation of endothelial cell proliferation	*ACVRL1 ENG GDF2 **KDR***
		GO:0003158	Endothelium development	*ACVRL1 ENG GDF2 **KDR** SMAD4*
		GO:0045446	Endothelial cell differentiation	*ACVRL1 ENG GDF2 **KDR** SMAD4*
		GO:0048762	Mesenchymal cell differentiation	*ACVRL1 ENG SMAD4 **SMO** TGFBR2*
		GO:0060485	Mesenchyme development	*ACVRL1 ENG SMAD4 **SMO** TGFBR2*
	Hypoxia response	GO:0001666	Response to hypoxia	*ACVRL1 **CHRNB2** ENG **KDR** SMAD4 TGFBR2*
		GO:0036293	Response to decreased oxygen levels	*ACVRL1 **CHRNB2** ENG **KDR** SMAD4 TGFBR2*
		GO:0070482	Response to oxygen levels	*ACVRL1 **CHRNB2** ENG **KDR** SMAD4 TGFBR2*
*Human Phenotype*	Cerebrovascular malformations	HP:0100026	Arteriovenous malformation	*ACVRL1 **BRCA2 DVL1** ENG **FANCC** GDF2 SMAD4*
		HP:0001009	Telangiectasia	*ACVRL1 **BRCA2** ENG **FANCC** GDF2 SMAD4*
		HP:0001048	Cavernous hemangioma	*ACVRL1 **AXIN1** ENG GDF2 SMAD4*
	Benign/malignant neoplasm	HP:0005306	Capillary hemangioma	*ACVRL1 **DVL1** ENG GDF2 **KDR** SMAD4*
		HP:0001028	Hemangioma	*ACVRL1 **AXIN1 DVL1** ENG GDF2 **KDR PRKN** SMAD4*
		HP:0100742	Vascular neoplasm	*ACVRL1 **AXIN1 DVL1** ENG GDF2 **KDR PRKN** SMAD4*
	Vessel dilatation	HP:0002624	Abnormal venous morphology	*ACVRL1 **AXIN1 BRCA2** ENG **EPHB2** GDF2 **NOTCH3** SMAD4*
		HP:0002619	Varicose veins	*ACVRL1 **AXIN1** ENG GDF2 **NOTCH3** SMAD4*
		HP:0004414	Abnormality of the pulmonary artery	*ACVRL1 ENG **FBN2** GDF2 **LTBP4** SMAD4 TGFBR2*
		HP:0004930	Abnormality of the pulmonary vasculature	*ACVRL1 **AXIN1** ENG **FBN2** GDF2 **LTBP4** SMAD4 TGFBR2*
		HP:0100659	Abnormality of the cerebral vasculature	*ACVRL1 **BRCA2** ENG GDF2 **NOTCH3 PKD1 PRNP** SMAD4 **SMO** TGFBR2 **WFS1***
		HP:0009145	Abnormal cerebral artery morphology	*ACVRL1 **BRCA2** ENG **FANCC** GDF2 **NOTCH3 PKD1** SMAD4 TGFBR2*
		HP:0011004	Abnormal systemic arterial morphology	*ACVRL1 **DNAH11** ENG **FBN2** GDF2 **NOTCH3 PKD1** SMAD4 TGFBR2*
*Mouse Phenotype*	Cerebrovascular malformations	MP:0006093	Arteriovenous malformation	*ACVRL1 ENG GDF2 **NOTCH3***
	Vessel dilatation	MP:0000259	Abnormal vascular development	*ACVRL1 **CD44 CTBP2** ENG **EPHA2 KDR HPGD MAML1 NOTCH3 PLXND1 LTBP1** SMAD4 **SMO SPP1** TGFBR2*
		MP:0001614	Abnormal blood vessel morphology	*ACVRL1 **CD44 CTBP2 DNAH11 DNAH5** ENG **EPHA2 FBN2** GDF2 **KDR PKD1 LMX1B LRP1 LTBP1 LTBP4 HPGD LRP2 MAML1 NOTCH3 PLXND1 SLIT2 NR1H4** SMAD4 **SPP1 TAB1** TGFBR2*
		MP:0003410	Abnormal artery development	*ACVRL1 ENG **NOTCH3 PLXND1 SMO KDR LTBP1** TGFBR2*
		MP:0004787	Abnormal dorsal aorta morphology	*ACVRL1 ENG **KDR** TGFBR2*
		MP:0000267	Abnormal heart development	*ACVRL1 **AXIN1 CTBP2 DNAH5** ENG **KDR MAML1 PKD1** SMAD4 **SMO** TGFBR2*
	Defects in embryo vasculogenesis	MP:0001719	Absent vitelline blood vessels	*ACVRL1 **CTBP2** ENG **KDR SMO** TGFBR2*
		MP:0003229	Abnormal vitelline vasculature morphology	*ACVRL1 **CTBP2** ENG **KDR SMO** SMAD4 TGFBR2*
*Disease*	Cerebrovascular malformations	C0003857	Congenital arteriovenous malformation	*ACVRL1 ENG **NOTCH3** SMAD4*
	Vessel disorders	C0334533	Arteriovenous hemangioma	*ACVRL1 ENG **NOTCH3** SMAD4*
		C0007820	Cerebrovascular Disorders	*ACVRL1 ENG GDF2 **NOTCH3 PKD1 RELN** SMAD4 **SPP1** TGFBR2*
		C0042373	Vascular Diseases	*ACVRL1 ENG **KDR NOTCH3 PRKN** SMAD4 **SPP1** TGFBR2*
		C0002940	Aneurysm	*ACVRL1 ENG **LRP1 LTBP4 PKD1** TGFBR2*

The table reports results obtained by ToppGene prioritization analysis giving as Training Gene Set *ACVRL1*, *ENG*, *GDF2*, *SMAD4*, and *TGFBR2*. According to the specific ontology, prioritized genes are shown in bold. Results are here summarized and grouped in relation to annotations. Data obtained for the single patient are available in Table S4. BMP: Bone Morphogenetic Protein; TGFBR: Transforming Growth Factor Beta Receptor.

**Table 3:** Variants affecting prioritized genes.

Locus	Gene	HGNC ID	SNP ID	Ensembl Transcript ID	Coding Sequence Change	Ensembl Protein ID	Protein Sequence Change
*1p22.3*	*CCN1* *	2654	rs765069158	ENST00000451137	c.G463C	ENSP00000398736	p.Gly155Arg
*1p36.12*	*EPHB2*	3393	rs142113032	ENST00000400191	c.847G>C	ENSP00000383053	p.Asp283His
*1p36.13*	*EPHA2*	3386	rs35484156	ENST00000358432	c.830C>T	ENSP00000351209	p.Ser277Leu
*1p36.33*	*DVL1*	3084	rs61735963	ENST00000378891	c.469G>A	ENSP00000368169	p.Ala157Thr
*1q21.3*	*CHRNB2*	1962	rs55685423	ENST00000368476	c.1191G>C	ENSP00000357461	p.Gln397His
*2p22.3*	*LTBP1*	6714	rs80163321 rs149319598	ENST00000404816	c. 2248 G>A c. 2410C>T	ENSP00000386043	p.Val750Ile p.Pro804Ser
*3p24.1*	*TGFBR2* *	11773	rs35766612	ENST00000295754	c.1159G>T	ENSP00000295754	p.Val387Leu
*3q22.1*	*PLXND1*	9107	rs137955512	ENST00000324093	c.2275C>T	ENSP00000317128	p.Pro759Ser
*4p15.31*	*SLIT2*	11086	rs115629108	ENST00000504154	c.4049G>A	ENSP00000422591	p.Ser1350Asn
*4q12*	*KDR*	6307	rs755067067	ENST00000263923	c.1990C>T	ENSP00000263923	p.Arg664Cys
*5q23.3*	*FBN2*	3604	rs28763954	ENST00000262464	c.976C>T	ENSP00000262464	p.Pro326Ser
*5q35.3*	*MAML1*	13632	rs113636707	ENST00000292599	c.569G>A	ENSP00000292599	p.Arg190His
*5q35.3*	*FLT4* *	3767	rs200763913	ENST00000261937	c.1133G>A	ENSP00000261937	p.Arg378His
*6p21.32*	*NOTCH4* *	7884	rs8192573	ENST00000375023	c.4037G>A	ENSP00000364163	p.Arg1346Gln
*7p13*	*CAMK2B*	1461	rs528355050	ENST00000395749	c.1577C>T	ENSP00000379098	p.Pro526Leu
*7q22.1*	*RELN*	9957	rs114684479 rs79499902	ENST00000428762	c.3477C>A c.5284G>A	ENSP00000392423	p.Asn1159Lys p.Val1762Ile
*7q36.1*	*GIMAP1* *	23237	rs1326399257	ENST00000307194	c.699G>A	ENSP00000302833	p.W233X
*9q34.11*	*ENG* *	3349	rs139398993	ENST00000373203	c.392C>T	ENSP00000362299	p.Pro131Leu
*10q26.13*	*CTBP2*	2595	rs1058301	ENST00000334808	c.387C>G	ENSP00000357816	p.Asp129Glu
*12q13.3*	*LRP1*	6692	rs113379328	ENST00000243077	c.7636G>A	ENSP00000243077	p.Gly2546Ser
*12q24.31*	*NCoR2* *	7673	Novel	ENST00000405201	c..2078G>T	ENSP00000384018	p.Glu693Ter
*15q22.1-q22.2*	*RNF111*	17384	rs142916216	ENST00000557998	c.888T>G	ENSP00000452732	p.Ile296Met
*16p13.3*	*AXIN1*	903	rs200741961	ENST00000262320	c.644C>T	ENSP00000262320	p.Ser215Leu
*17p11.2*	*MAP2K3*	6843	rs33911218 rs36047035	ENST00000342679	c.118C>G c.164G>C	ENSP00000345083	p.Pro40Ala p.Arg55Thr
*19p13.12*	*NOTCH3*	7883	rs141320511	ENST00000263388	c.4552C>A	ENSP00000263388	p.Leu1518Met
*19q13.2*	*LTBP4*	6717	rs35809725	ENST00000308370	c.4499A>T	ENSP00000311905	p.Tyr1500Phe
*22q13.1*	*TAB1*	18157	rs536084162 rs140879164	ENST00000216160	c.19A>C c.560G>A	ENSP00000216160	p.Ser7Arg p.Arg187His

For each variant, chromosomal band, gene name, HUGO Gene Nomenclature Committee gene name, variant ID, transcript and protein reference IDs of the Ensembl Genome Browser, coding sequence, and amino acid position changes are reported. * Loci not output by the ToppGene tool.

**Table 4:** Anamnesis data of the patients enrolled in the study.

Patient	Sex	Age (Years)	Age of Onset (Years)	Symptoms	Lesion Number	Spetzler–Martin Grading
***AVM1***	M	14	12	Intracerebral hemorrhage following AVM rupture	1—parietal left area	2
***AVM2***	M	31	18	Tremor of the left leg, diffuse tremor, seizures	1—front-parietal left area (not bleeding)	2
***AVM3***	F	32	29	Dizziness, tinnitus, seizures nausea right hemiparesis visus reduction	1— parietal left area	3
***AVM4***	F	8	At birth	Drowsy status, finalistic limb movement	1—proliferative microangiopathy, central left area	3
***AVM5***	M	7	5	Sudden headache, vertigo, seizures	1—anterior-parietal paramedian right area	2

The patients are named as mentioned in the text (AVM1–5). Gender, age, and clinical features are reported for each patient. AVM: Arteriovenous malformation. The number (1–5) indicates the sample.

## Supplementary Materials

Supplementary materials are available at https://www.mdpi.com/1422-0067/21/12/4321/s1. Table S1: AVM Sample Reports of WES Analysis, Table S2: Selected Variants, Table S3: ClueGO Pathways, Table S4: ToppGene Results. Table S5.: STRING Results.
